# Graphic Illustrations of Abortion and the Diseases of Menstruation

**Published:** 1833-07-01

**Authors:** 


GRAPHIC ILLUSTRATIONS OF ABORTION AND THE DISEASES OF MENSTRUATION. Consisting of 12 Plates from Drawings Engraved on Stone, and Coloured by Mr. J. Perry, and two Copper-plates from the Philosophical Transactions, Coloured by the same Artist. The whole representing 45 specimens of Aborted Ova, and adventitious productions of the Uterus, with Preliminary Observations, Explanations of the Figures, and Remarks, Anatomical and Physiological. By *A. B. Granville*, MD., FRS., FLS., FGS., FRAS., &c. &c.

COLOURED lithographs, if ably executed, are now allowed to be the best mode of conveying an accurate idea of anatomical subjects; but, unfortunately, our artists, on this side of the channel, have hitherto failed to come up to the level of their rivals on the Continent. This series of drawings, executed by Mr. J. Perry, of Warren Street, under the immediate superintendence of Dr. Granville, will, we firmly believe, prove that lithographic representation has, at last, on British soil, equalled, if not excelled, any thing of the kind in foreign countries. This is no mean praise—but Mr. Perry is fairly entitled to it. The anatomical preparations, in every case, were placed before the artist, by Dr. Granville, and explained and demonstrated to him. The result is, that, in the course of six years, twelve plates, containing upwards of forty anatomical figures, have been produced, lithographed and coloured by Mr. Perry, rivalling the most successful efforts of French, German, or Italian artists.

In respect to Dr. Granville himself, it appears that he has selected such specimens only as he considered likely to illustrate the more interesting points of physiology as connected with human generation, and which might assist in unravelling the many practical difficulties that beset that thorny question. He has also chosen those which, with a very few exceptions, have not hitherto been published, as far as we know.

Hunter and Soemmering, indeed, have given us splendid engravings of the gravid uterus, and of the human embryo; but they are nearly inaccessible, from price and scarcity, to the professional reader. Even when possessed, they lose the value of colouring; and are inferior, in point of art, to the present lithographs.

"With one or two exceptions, the representations in Soemmering's work are simply those of the fœtus in its progressive advancement from the third week after fecundation, to the fifth or sixth month of gestation. Those representations have no reference to the whole ovum at those several periods of fecundation: and they are not coloured. Still they are, as he has stated, superior as a work of art to any that had appeared before. Soemmering tells us that he selected for his plates the best specimens of the human embryo, the best draughtsman, and the most skilful engraver; and that when he compared those plates with those of Trioenius, Albinus, Wrisberg, Hunter, and Denman, in order that he might better understand, explain, and perhaps correct his own: '*magnopere delectabatur*,' to find on such a comparison, that the superiority of his plates was not inconsiderable. Well, then, let the reader who has the means of doing so, turn to the two folio plates of Soemmering, and then direct his attention to the fourteen plates contained in the present volume, and I will abide by their decision whether or no, in all such delineations as relate to either parallel or identical subjects in the two publications, the palm of superiority (in every circumstance of design, precision, and execution,) which Soemmering claimed for *his* publication, ought not to be yielded by him to *another* sent forth under the advantage of recent improvements in drawing, and a newly invented art admirably adapted for such imitations of nature. Independently of which advantages there is to be added the charm of colour—a circumstance which so greatly embellishes, without disfiguring, truth." xiii.

That such a series of representations as is here given, must be highly useful to the profession at large, admits of no cavil or doubt; and we can only say that the moment we saw the work, we subscribed to it, from a sense of duty to industrious merit, though it is probable that, in the common course of things, such subscription was unnecessary, as far as pecuniary matters are concerned. We cannot let this opportunity pass, without reminding our readers that, in all works of this nature, there is necessarily a great outlay of money, independent of labour, and consequently that, unless patronage is bestowed on the author he is left a great loser in pocket, by the effort to advance medical science and lay information before the public. We are aware, indeed, that a vast majority of the profession are unable to purchase expensive works, but these should propose and urge the purchase of such works by medical societies and book-clubs, now so universally diffused over town and country.

Under the head Prolegomena, and in 102 Sections or propositions, our author has concentrated, as it were, the elementary principles of all that is known respecting the origin, formation, progress, and metamorphoses of the fœtus, and its connexion with the mother. In these Dr. G. has added his quota of new information to the general stock, especially as connected with the important subject of abortion. Dr. G. limits his observations on intro-uterine existence, to the first five or rather four months, because, after that period the various phenomena of fœtal gestation are so uniform as to afford but little matter of interest to the physiological inquirer. These propositions being, as it were, a dense analysis of volumes of experiments and observations, it is impossible for us to attempt an analysis of them; yet we must offer our readers some specimens of these Prolegomena, in order that they may judge both of their matter, and the manner in which they are constructed.

"13. CONCEPTION, or that result which follows sexual congress, in virtue of which one, or more individuals, of the same species is called into being, takes place in the ovarium of women. This is doubted by Meckel and others, who look upon all cases of ovaric gestation (see Plates IX. and X. A. and B.) as mere accidents, and as only proving that if conception has not before taken place in the womb, it *may* take place in some other part connected with it; but the point has been set at rest by the more recent experiments and microscopical observations of Professor BOER, of Kœnigsberg. I adopt his conclusions. Their correctness is corroborated by the interesting experiments of Prevost and Dumas, although these experimenters admit not that fecundation takes place in the ovarium.

14. The intended receptacle of the embryo is the OVULUM. An ovulum exists in all the vesicles of Graaf, which the ovarium of a woman, who has reached maturity, contains.

15. Viewed by means of a powerful microscope, the ovulum is found to consist of a small yellow spherical body, placed within the vesicula Graafiana, with the upper portion of which it is, internally, in contact; so that it does not float freely in the liquid of that vesicle. This contact becomes more and more intimate as the ovulum enlarges, when that part of the capsule of the vesicle which lies over it becomes, in a correspondent degree, thinner.

16. At first, the little yellow body, being rather opaque, is distinctly seen even without a magnifying glass; but as it advances, it becomes more transparent and, consequently, less distinguishable.

17. This little yellow body is a minute spherical mass, with a roughish or slightly granular surface, and is hollow. Its parietes are thick, around them is an envelope of a much thinner texture, which is distinctly seen, owing to a small space lying between it and the surface of the little yellow body, which space is filled with a fluid substance of a peculiar nature.

18. When FECUNDATION takes place, that part of the vesicle of Graaf to which adheres, internally, the ovulum, bursts, and the ovulum escapes with its external envelope, together with a small portion of the liquid peculiar to the Graafian vesicle, and thus it passes into the fallopian tube.

19. Independently of the external envelope, and within it, the microscope has detected, after fecundation, the existence of another covering, completely investing the little spherical yellow body.

20. The ovulum has been traced, after fecundation, into the cavity of the womb, where the external covering (16) becomes what Boer has called 'the cortical membrane,' (*cortex ovi* of the present work,) improperly considered as a uterine production by preceding writers, and denominated the *reflected* caducous or deciduous membrane.

21. The more intimate covering of the yellow body of the ovulum, that which closely invests its surface, and appears only after fecundation, (19) is afterwards changed into what has been denominated the shaggy chorion: my observations and my plates shew this. Boer, however, professes not to know what becomes of it during the progressive introuterine development of the ovulum.

22. The hollow and spherical yellow body of the ovulum corresponds with the yelk or *vitellus* of the ovum of oviparous animals, and from it all the other several parts of the fœtiferous ovum are derived or formed, as gestation advances, and a progressive development of the parts takes place, from within, without.*"

* "Professor Boer, who fills the Chair of Zoology at Kœnigsberg, is a man of undoubted veracity, a keen and accurate observer, and has been engaged for many years in the investigation of that most interesting function—reproduction —in mammiferous animals. He made a great number of minute and extremely delicate experiments and microscopical observations on animals and the ovaria of women, which led him to the conclusions I have embodied in my propositi-

"26. The cortical membrane is destined to be absorbed during the first months of utero-gestation, thus exposing the next membrane to the contact of the uterine lining (decidua,) with which a connexion takes place in that part where the placenta is to be formed. In that part, however, the cortex ovi is never altogether obliterated, but only made thinner; and, in process of time, it is converted into a mere pellicular envelope, which not only serves to divide the filiform vessels of the chorion into groups or cotyledons in order to form the placenta, but also covers all over those cotyledons or groups of vessels. I have called this the *membrana propria.**

27. While the process or metamorphosis of the ovulum noticed by Boer takes place in the ovarium, in consequence of fecundation, the cavity of the womb does not remain idle, but forthwith sets about weaving for itself a general lining—a sort of pseudo-textile membrane—which extends all over the cavity, descends partly into the cervix, and is *often* (not necessarily always,) projected even into a great portion of the fallopian tubes.

28. This adventitious lining of the cavity of the womb is formed quite independently of the presence of the ovum, for it has been found in most cases of devious gestation, where the fœtus was *extra muros uteri,* and has been found advanced in its progress of formation, while the ovulum was, as yet, on its way through the fallopian tube after fecundation. (Haller, Lobstein, Velpeau, Meckel, Pockels.)"

"31. It is probable that the decidua consists of two laminæ, inasmuch as we always find it with one surface perfectly smooth and the other rough. If so, they are most intimately connected. It is at least one-twentieth of an inch in thickness during the first five or six weeks of uterofœtation, when its tissue is found to be more knotty, coarse, and full of short threads, (not unlike a very ordinary mat,) than a purely membranaceous or cloth-like lining would be. It is not until a more advanced period of gestation that the decidua becomes distinctly membranaceous, in which state it lines the entire cavity of the uterus."

"34. As soon as the Ovulum has departed from its vesicular nest in the Ovarium, the cavity which remains begins to fill up with a yellow substance, different in texture from the surrounding tissue of the Ovarium, and having, generally, a radiated centre of a whiter colour. This is the *corpus luteum.*

35. The presence of *corpora lutea* in the Ovarium of women, is always an indication that as many ovula have escaped from that organ; but it is not necessarily an evidence that the individual has been impregnated, as ovula have escaped without the congress of the two sexes.

---

ons, and which he forwarded in a Latin epistle, entitled 'De Ovi mammalium et hominis genesi,' to the Imperial Academy of St. Petersburgh, with a plate, carefully engraved, representing all the details above alluded to. These he afterwards, and within the last four years, enlarged upon very considerably in a subsequent publication."

* "If the reader can procure a placenta which has been thrown off immediately after the birth of the child, without any effort, and cleaning it of all coagula from off the surface which lay next to the uterus, by careful maceration and washing, he will afterwards introduce a small quill or pointed tube into one of the arteries of the navel-string, and blow strongly into it, he will find that the air raises upon that surface, to various degrees of puffiness, a very delicate pellicular covering, through which none of the air can escape, unless through an accidental laceration. I have often made the experiment, which I used to relate to my class in my lectures on midwifery many years ago. Lauth, of Strasburgh, has stated the same thing; so had Ruysch long since, and others, proving at once that there is not a direct communication with the mother from the fœtus."

36. It is inaccurate, therefore, to state that a woman has been pregnant because a *corpus luteum* has been found in one of the Ovaria after death, or to calculate the number of children she has borne from the number of *corpora lutea* so detected. *Corpora lutea* have been found in the Ovaria of very young girls, of unmarried women of the strictest virtue, in newly-born female infants, and lastly, in sterile animals, such as mules." vi.

The ovulum, on entering the womb, is about the size of a small pea; but the interval of time, between fecundation and its entrance into the uterus, is not exactly known. On the 14th day the ovulum is about the size of a Spanish nut. The chorion is surrounded by a thick membrane—the cortex ovi. After being safely lodged in utero, the ovulum continues to grow on its own life principle, for a while, until its connexion with the mother through the medium of the deciduous membrane, which becomes, as it were, an additional covering to the ovulum. The growth of the ovulum causes the cortex to burst, as happens with the cortex of certain seeds, and with the outer shell of the ova of some oviparous animals.

" On the cortex bursting, the lanuginous or fibrillous membrane within it (21) is exposed, when the fibrils will forthwith entwine themselves with the flocculi of the decidua, and thus the Ovulum fastens itself to the uterus by one or more contiguous points.

The membrane having these fibrils on its surface, has been called the chorion —and from the circumstance that these fibrils, both before the cortex which lies over them has burst, as well as afterwards, serve to promote the nourishment of the fœtus, I have styled it, the nutritive membrane or involucrum of the fœtus. It has been so considered by Ruysch, who calls the villous side of the chorion, '*succosa nutritioni fœtus inserviens.*'" viii.

This nutritive membrane is bifoliated—perhaps trifoliated. The internal surface of the chorion is vascular, as is proved by its diseases, chiefly of an inflammatory character, ending in thickening of texture. Also by injections.

" These facts, demonstrative and corroborative of the vascularity of the chorion, (45, 46, 48, 49,) explain and account for the reality of that self-existing life-principle inherent in the fecundated Ovum (42), which detaches it from its nest (vesicula Graafiana), enables it to travel through the tube, to grow or expand while thus travelling, and to maintain that same power of growth and development for a short time after its reception into the womb, until its final and effectual implantation on the maternal stock (uterus.)" viii.

The same holds good with respect to the amnion, or inner transparent membrane of the ovum, as proved by morbid anatomy. And if a vascular membrane, there is no difficulty in conceiving it to be a secreting one also —hence the source of that particular fluid, the liq. amnii, in which the embryo is suspended.

Dr. Granville thinks it probable that the embryo lives throughout the whole period of utero-gestation, by virtue of its own life-principle.

" When the Ovulum has made good its fastening to the adventitious lining of the womb (decidua), the circulation of the blood in it is as yet imperfect. The Ovulum does not—cannot—receive the blood of the mother. How could such a gossamer-like being, organized as the Ovulum has been proved to be, during the first days after fecundation, be made a part of so impetuous a torrent as the circulation of the blood of the mother, without instant destruction to the produce of conception? No. The blood of the embryo is first formed within itself. (Prevost, Home, Magendie, Adelon, Serres, Rolando.)" x.

The new BEING passes through two striking metamorphoses previously to the enjoyment of extra-uterine life—the embryonic, and the fœtal states. The latter begins at the moment when the new being is grafted on the maternal womb, and continues till parturition. In the former, or embryonic state, it is without any communication, direct or indirect, with the mother—still less so, with the external world. This state persists for about two weeks after fecundation, during which period the embryo derives its nutrition from the cortical membrane of the ovum. Up to the second month, the growth of the embryo is slow—it is accelerated during the third—slackens again in the fourth and fifth months; between which and the last month the increase is more rapid.

Until within these few years it was supposed that the nervous system was formed first; but recent examinations have proved that the vascular system takes precedence in the nisus formativus. The spinal marrow appears before the brain, and the cerebrum before the cerebellum.

"The blood is formed independently of the heart, and appears at two distinct points from it, and acquires a motion independently of it. (Prevost, Dumas, Baer.) The veins are formed first—next, the heart—lastly, the arteries, &c. (French Physiologists.) The arteries are, by an Italian physiologist, said to be the first to appear. (Rolando.)" xii.

The intestinal canal is the first part of the digestive apparatus that appears.

A considerable space is occupied in delineating the connexion between the placenta and the uterus, which we must pass over, with the exception of an extract or two.

"86. The decidual vessels derive their fluid from the uterine vessels. The arteries which convey uterine blood to the decidual vessels, are tortuous and very small; they are the adventitious produce of the *membrana propria* of the womb acting under the influence of a peculiar stimulation which produces the decidual membrane, as inflamed surfaces produce organized exudations. Though the latter be formed in the uterus, even when the embryo is lodged, by aberration, in some other part of the abdomen, its presence must not be deemed, on that account, unessential to the embryo; for a vascular membrane, as nearly alike to it in texture as can be, has invariably been found to connect, by blood-vessels, the embryo to some vital part nearest to where that embryo has been casually deposited, that part having, at the same time, its circulation and vascularity greatly increased, and becoming, in fact, the parent of the connecting vascular membrane in question.

87. Nothing proves more distinctly, (it might be said almost to demonstration,) the accuracy of the views (82, 83, 84, 85,) which tend to establish the fact of a vascular communication between the arterio-venous system of the mother and the placenta (by intermediate decidual circulation) and to shew the fallacy of those who deny such a communication, than the very phenomenon just noticed (86). Here morbid anatomy again comes to the assistance of normal anatomy and physiology, and affords evidence which is not liable to the errors that have been unjustly affixed to experimenters with injected fluids. Of the many examples that might be quoted in support of this proposition, the one which is stamped with the authority of Lallemand may be selected as the most striking.* In a case of ventral aberrant fœtation, which had proceeded to the end of the

* "Observations relatives à la Génération. Par F. Lallemand. Paris, 1818."

sixth month, before it destroyed the patient, a vascular and tomentous membrane had been formed on the surface of the peritoneum, to which adhered the regular placenta and chorion of the fœtiferous ovum. This membrane resembled in every respect the decidua at six months—it was thicker, and more red and vascular where the placenta was adherent than any where else. 'Vessels as visible as those of the inflamed conjunctiva,' observes the author, 'passed from the highly-injected peritoneum, opposite the placenta, into the membrane which lay between them; while other vessels from the placenta reached as far as the same membrane, and were lost in it where they probably anastomozed by their very minute terminal ramifications.' (Lallemand.) The conclusion which this really eminent physiologist and good man has come to, upon this subject, is striking, and truly to my purpose. 'The decidua,' says he, 'has no other function to perform than that of serving as a capillary system, intended to be the medium of communication between the blood-vessels of the mother and those of the fœtus.' (page 21.)

88. It is possible that the venous blood of the decidual vessels may be returned through the great uterine sinuses, the large open orifices of which, covered with an almost valvular flap, have been described by the best anatomists, as being applied to the surface of the decidual placenta. Magendie* thus states his opinion on this subject. 'In women large openings, which communicate with the uterine veins, are observed on that part of the uterus to which the placenta adheres; but it is not clear whether these venous orifices are destined to absorb the blood of the fœtus, or to suffer that of the mother to escape on the surface of the placenta. I am inclined to admit the latter idea—but no proof whatever exists of its correctness." (page 554.) xvii.

The circulation of the blood in the ovum appears to be independent of that of the mother—the embryo creating its own blood, and supporting its own existence. Yet its blood requires changes, and those changes are produced through the influence of the mother's blood. The placenta appears to be the organ through which this change is effected.

"The decidual vessels receive the arterial blood of the mother. This is spread over a very considerable surface of tubular structure, which being, in its distribution, made to come in apposition with the infinite ramifications of the umbilical placental vessels, at innumerable points, (like the inspired air distributed through the bronchial passages is made to come in apposition with the myriads of vascular rami of the lungs); the required changes in the blood of the fœtus are produced, just as the changes called for in the pulmonic blood, are produced by the peculiar arrangement of that part of the animal economy. When the arterial blood of the mother has produced the desired effect on that of the fœtus —it is returned by the decidual veins to the uterine sinuses applied, like absorbing mouths, to the surface of the decidua, when it enters into the general venous system of the mother. (Magendie; Personal Observations.)" xviii.

Our author is inclined to think that some degree of respiration goes on in the fœtus, as a means of facilitating growth and supporting life. This opinion is entertained by Geoffroy St. Hilaire and Müller. The presence of air has been detected in the amnionic fluid by Lassaigne, and by Dr. Granville himself. The process of respiration, if it take place at all, is supposed to be effected by the cutaneous pores, as in aquatic insects.

"It is of the utmost importance to bear in mind the great distinction which

---

* "Precis Elementaire de Physiologie, 2d. edit. Paris, 1825.

exists between the independence of the fœtus, *quoad* life, and its dependence, *quoad* nutrition, in respect to the mother. The former state is secured by a total separation of the two circulations (maternal and fœtal). The latter by the close reciprocal contact of the organs of those circulations. Thence is it that we find the fœtus to live on, notwithstanding that its connexion with the mother has been partially and sometimes even wholly, severed;—while on the other hand we cannot help admitting that, albeit this independence, the influence of the mother over the fabric of her offspring is unquestionable." xx.

The foregoing particulars have been picked out of Dr. Granville's Prolegomena, rather as bricks from a building, to exhibit specimens of the materials, than as affording any idea of the edifice itself. The great body of the volume is occupied by the plates—the descriptions of those plates—and notes, physiological, pathological, and practical. These will require another notice; mean time, we cannot too earnestly advise every practitioner who desires to make himself thoroughly acquainted with the subject of this volume, to possess himself of the work.

